# ﻿Arthrocatenales, a new order of extremophilic fungi in the Dothideomycetes

**DOI:** 10.3897/mycokeys.108.128033

**Published:** 2024-08-22

**Authors:** Marcin Piątek, Monika Stryjak-Bogacka, Paweł Czachura

**Affiliations:** 1 W. Szafer Institute of Botany, Polish Academy of Sciences, Lubicz 46, PL-31-512 Kraków, Poland W. Szafer Institute of Botany, Polish Academy of Sciences Kraków Poland

**Keywords:** Dothideomycetes, molecular phylogeny, new combination, new family, new order, taxonomy

## Abstract

The widely treated order Capnodiales is one of the most important orders in the class Dothideomycetes. Recently, the order Capnodiales s. lat. was reassessed and split into seven orders (Capnodiales s. str., Cladosporiales, Comminutisporales, Mycosphaerellales, Neophaeothecales, Phaeothecales and Racodiales) based on multi-locus phylogeny, morphology and life strategies. In this study, two *Arthrocatena* strains isolated from sooty mould communities on the leaves of *Tiliacordata* and needles of *Pinusnigra* in southern Poland were analyzed. Multi-locus phylogenetic analyses (ITS-LSU-SSU-*rpb2*-*tef1*) along with morphological examination showed that they belong to *Capnobotryellaantalyensis*, which represents a sister taxon to *Arthrocatenatenebrosa*. *Capnobotryellaantalyensis* is a rock-inhabiting fungus described from Turkey. The following new combination is proposed: *Arthrocatenaantalyensis*. Phylogenetic analyses also showed that *Arthrocatena* and related genus *Hyphoconis*, both known previously only from rocks, form a sister lineage to orders Cladosporiales and Comminutisporales. The new order Arthrocatenales and new family Arthrocatenaceae are proposed to this clade. Representatives of this order are extremophilic fungi that live on rocks and in sooty mould communities.

## ﻿Introduction

The order Capnodiales in the wide sense (s. lat.) is one of the most important orders in the class Dothideomycetes. It contains thousands of species growing in all areas of the world, the majority of known environments, including most extreme ones, and showing diverse nutritional modes and life strategies (Schoch et al. 2006; Muggia et al. 2008; Crous et al. 2009b, 2013a; Bensch et al. 2012; Groenewald et al. 2013; Hujslová et al. 2013; Quaedvlieg et al. 2013, 2014; Egidi et al. 2014; Wijayawardene et al. 2014; Isola et al. 2016; Duarte et al. 2017; Videira et al. 2017; Gleason et al. 2019; Abdollahzadeh et al. 2020; Czachura et al. 2021; Piątek et al. 2023). This wide concept of the order Capnodiales was recently reassessed by Abdollahzadeh et al. (2020) who split it into seven orders (Capnodiales s. str., Cladosporiales, Comminutisporales, Mycosphaerellales, Neophaeothecales, Phaeothecales and Racodiales) based on multi-locus phylogeny, morphology and life strategies. The redefined order Capnodiales s. str. includes species that are almost exclusively sooty moulds while the remaining orders comprise genera and species generally encompassing other nutritional modes and ecologies (Abdollahzadeh et al. 2020), although Cladosporiales and Mycosphaerellales include some species isolated from sooty mould communities too (e.g., Friend 1965; Flessa et al. 2012, 2021; Flessa and Rambold 2013; Crous et al. 2023a, 2023b; Piątek et al. 2023). The phylogenetic placement of several genera and families within the Capnodiales s. lat. is still unresolved (e.g., Quaedvlieg et al. 2014; Ismail et al. 2016; Abdollahzadeh et al. 2020; Pereira and Phillips 2020). This refers, for example, to two phylogenetically closely related genera *Arthrocatena* and *Hyphoconis* that accommodate single species, *Arthrocatenatenebrosa* and *Hyphoconissterilis* described as rock-inhabiting fungi from Italian Alps and Mediterranean Spain, respectively (Egidi et al. 2014; Crous et al. 2019a).

Sooty moulds are epiphytes associated with honeydew or sweet plant exudates occurring on the leaves/needles of woody plants (Hughes 1976). Although many species of sooty moulds reside in the order Capnodiales s. str. (Abdollahzadeh et al. 2020) they are also known in other orders and families of the classes Dothideomycetes and Eurotiomycetes (e.g., Friend 1965; Flessa et al. 2012, 2021; Chomnunti et al. 2014; Piątek et al. 2023). Sooty moulds form a multi-species assemblages (Hughes 1976; Hughes and Seifert 2012; Flessa et al. 2012, 2021) containing even 243 species (OTUs) (Dhami et al. 2013) of which many probably remain undescribed.

Fungi isolated from sooty mould communities are sometimes phylogenetically related to rock-inhabiting fungi. Such a relationship was mentioned in the orders Capnodiales (s. lat.) and Chaetothyriales (Chomnunti et al. 2014) and in the genera *Lapidomyces* (order Mycosphaerellales) and *Rachicladosporium* (order Cladosporiales) (Crous et al. 2023b; Piątek et al. 2023). *Capnobotryellarenispora* has been described as a sooty mould associated with other sooty mould *Capnobotrysneesii* growing on *Abiesveitchii* branches in Japan (Sugiyama and Amano 1987) and later found on roof tiles that is a habitat resembling rocks (Titze and de Hoog 1990).

In a recent survey of sooty mould communities occurring on ornamental woody plants in urban environments in southern Poland we isolated two strains that were assigned to the genus *Arthrocatena* based on initial ITS rDNA sequencing. This study aims to identify isolated *Arthrocatena* strains using morphology and multi-locus phylogenetic analyses and to clarify the phylogenetic placement of the genera *Arthrocatena* and *Hyphoconis* within Capnodiales s. lat.

## ﻿Materials and methods

### ﻿Isolates

Fungal isolates studied here were obtained from sooty mould communities on ornamental woody plants cultivated in municipal greenery in cities of southern Poland. The initial isolations were made on malt extract agar (MEA – Blakeslee’s formula), potato dextrose agar (PDA), and rose bengal agar (RBC). The details of microbiological media and method of initial isolation are described in Piątek et al. (2023). Dried specimens obtained from cultures are stored in the fungal collection of the W. Szafer Institute of Botany, Polish Academy of Sciences, Kraków (KRAM F). Cultures are deposited in the culture collection of the Westerdijk Fungal Biodiversity Institute (CBS) and in the W. Szafer Institute of Botany, Polish Academy of Sciences, Kraków.

### ﻿Morphological analyses

Macroscopic characteristics were documented using 4-week-old colonies growing on MEA and PDA incubated at 25 °C. Microscopic characteristics were examined under a Nikon Eclipse 80i light microscope using slide cultures on PDA incubated at 25 °C, after approximately one month growth (Crous et al. 2019b). The disintegration of chains of arthroconidia was observed on MEA cultures. The microscopic structures were measured and photographed using NIS‐Elements BR 3.0 imaging software. Growth at 15 °C and 25 °C on MEA and PDA was assessed by measuring the colony diameter after 2 weeks and 4 weeks.

### ﻿DNA isolation, amplification and sequencing

DNA was extracted using DNeasy® Plant Mini Kit (Qiagen, Germany), according to the manufacturer’s protocol. Four loci were amplified: ITS1‐5.8S‐ITS2 rDNA (= ITS), fragment of the large subunit rDNA (28S D1–D2 = LSU), the small subunit rDNA (18S = SSU) and protein-coding gene – partial DNA-directed RNA polymerase II second largest subunit (*rpb2*). The following primer pairs were used for amplification: ITS1–ITS4 for ITS (White et al. 1990), LSU1Fd–LR5 for LSU (Vilgalys and Hester 1990; Crous et al. 2009b), NS1–NS4 for SSU (White et al. 1990), and fRPB2-5F–fRPB2-7cR for *rpb2* (Liu et al. 1999). Polymerase chain reactions were performed in a reaction mixture prepared as described in Piątek et al. (2023). ITS and LSU were amplified as described by Czachura et al. (2021). Amplification of SSU was performed with initial denaturation at 94 °C for 3 min followed by 35 cycles of denaturation at 94 °C for 45 sec, the annealing of primers for 30 sec at 52 °C, the elongation at 72 °C for 1 min and the final extension at 72 °C for 10 min. Amplification conditions for *rpb2* were set as follows: an initial denaturation at 94 °C for 3 min, followed by 35 cycles of denaturation at 94 °C for 60 sec, the annealing at 54 °C for 90 sec, the elongation at 72 °C for 2 min and the final extension at 72 °C for 10 min. Amplicons were visualized and verified by gel electrophoresis on 1% agarose gel. Subsequently, the PCR products were enzymatically purified using an Exo-BAP Mix (EURx, Poland) and sequenced bidirectionally by Macrogen Europe B.V. (Amsterdam, the Netherlands). Obtained sequences were assembled and trimmed in Geneious Prime 2020.0.4. Consensus sequences were deposited in the NCBI’s GenBank nucleotide database (https://www.ncbi.nlm.nih.gov/genbank/).

### ﻿Phylogenetic analyses

The affinity of the isolated strains was first checked in the NCBIs GenBank nucleotide database using the megablast search tool (Zhang et al. 2000). To resolve phylogenetic placement of the isolated strains, the concatenated ITS-LSU-SSU-*rpb2*-*tef1* alignment was assembled. LSU, *rpb2* and *tef1* sequences of species and strains used for phylogenetic reconstructions were mostly selected from study of Abdollahzadeh et al. (2020). ITS and SSU sequences of the same species and strains were additionally added to the dataset. Finally, the sequences of *Arthrocatenatenebrosa*, *Capnobotryellaantalyensis* and *Hyphoconissterilis* revealed as most closely related to sequences of analyzed strains were also added to the dataset (Table 1).

**Table 1.** List of species, with country of origin, host/substrate, strain, GenBank accession numbers and references, used in phylogenetic analyses.

Species	Country	Host/substrate	Strain	GenBank acc. no.					References
				ITS	LSU	SSU	*rpb2*	*tef1*	
*Aeminiumludgeri*	Portugal	limestone	E14	MG938062	MG938288	–	–	–	Trovão et al. 2019
*Aeminiumludgeri*	Portugal	limestone	E8	MG938056	MG938284	–	–	–	Trovão et al. 2019
*Aeminiumludgeri*	Portugal	limestone	E12	MG938054	MG938286	–	–	–	Trovão et al. 2019
*Amycosphaerellaafricana*	South Africa	leaves of *Eucalyptusviminalis*	CBS 680.95	MH862549	KF902048	–	–	–	Quaedvlieg et al. 2014; Vu et al. 2019
*Amycosphaerellakeniensis*	Kenya	leaf litter of *Eucalyptusgrandis*	CBS 111001	MF951290	GQ852610	NG_062384	MF951433	–	Crous et al. 2009b, 2009c; Videira et al. 2017
*Arthrocatenaantalyensis* (syn. *Capnobotryellaantalyensis*)	Poland	sooty mould community on *Tiliacordata*	CBS 150720	OR096278	OR096282	OR096280	OR096699	–	this study
*Arthrocatenaantalyensis* (syn. *Capnobotryellaantalyensis*)	Poland	sooty mould community on *Pinusnigra*	CBS 150721	OR096279	OR096283	OR096281	OR096700	–	this study
*Arthrocatenaantalyensis* (syn. *Capnobotryellaantalyensis*)	Turkey	marble	MA 4659	AJ972854	–	AJ972854	–	–	Sert et al. 2007
*Arthrocatenaantalyensis* (syn. *Capnobotryellaantalyensis*)	Turkey	marble	MA 4775	AJ972860	–	AJ972860	–	–	Sert et al. 2007
*Arthrocatenatenebrosa*	Italy	rock	CCFEE 5413	NR_144971	NG_056969	NG_061095	–	–	Ruibal et al. 2009; Egidi et al. 2014
*Aureobasidiumpullulans*	France	*Vitisvinifera*	AFTOL-ID 912	–	DQ470956	DQ471004	DQ470906	DQ471075	Spatafora et al. 2006
*Austroafricanaassociata*	Australia	*Protealepidocarpodendron*	CBS 112224	DQ302968	KF901827	GU296200	–	GU349025	Crous et al. 2006; Schoch et al. 2009; Quaedvlieg et al. 2014
*Batcheloromycessedgefieldii*	South Africa	*Protearepens*	CBS 112119	NR_137012	KF937222	–	–	–	Crous et al. 2008; Quaedvlieg et al. 2014
*Capnobotryellarenispora*	Japan	*Capnobotrysneesii*	CBS 214.90	NR_121295	NG_058782	NG_070856	–	–	Hambleton et al. 2003; Scott et al. 2007; Crous et al. 2009b
*Capnodiumalfenasii*	Brazil	*Tabebuia* sp.	CBS 146151	MN749233	MN749165	–	MN829260	MN829346	Abdollahzadeh et al. 2020
*Capnodiumblackwelliae*	USA	*Myrtuscommunis*	CBS 133588	MN749235	MH878118	–	GU371743	GU349054	Schoch et al. 2009; Vu et al. 2019; Abdollahzadeh et al. 2020
*Capnodiumcoartatum*	Thailand	*Psidium* sp.	MFLUCC 10-0069	–	JN832614	JN832599	–	–	Chomnunti et al. 2011
*Capnodiumcoffeae*	Zaire	*Coffearobusta*	CBS 147.52	MH856967	GU214400	DQ247808	KT216519	DQ471089	Spatafora et al. 2006; Crous et al. 2009b; Ismail et al. 2016; Vu et al. 2019
*Capnodiumcoffeicola*	Thailand	*Coffea* sp.	MFLUCC 15-0206	–	KU358920	–	–	–	Hongsanan et al. 2015b
*Capnodiumgamsii*	Sri Lanka	unknown leaf	CBS 892.73	MN749237	GU301847	–	GU371736	GU349045	Schoch et al. 2009; Abdollahzadeh et al. 2020
*Capnodiumneocoffeicola*	Thailand	*Coffeaarabica*	CBS 139614	MN749242	MN749172	–	MN829267	MN829353	Abdollahzadeh et al. 2020
*Capnodiumparacoffeicola*	Thailand	*Coffeaarabica*	CBS 139616	MN749244	MN749174	–	MN829269	MN829355	Abdollahzadeh et al. 2020
“*Capnodium*” *salicinum*	Indonesia	*Bursariaspinosa*	CBS 131.34	MH855469	EU019269	DQ677997	KT216553	DQ677889	Schoch et al. 2006b; Crous et al. 2007a; Ismail et al. 2016; Vu et al. 2019
*Cercosporabeticola*	Italy	*Betavulgaris*	CBS 116456	NR_121315	DQ678091	NG_062715	KT216555	DQ677932	Groenewald et al. 2005; Schoch et al. 2006b; Ismail et al. 2016
*Cercosporellavirgaureae*	South Korea	*Erigeronannuus*	CBS 113304	GU214658	KF251805	GU214658	KX348051	–	Crous et al. 2009b; Verkley et al. 2013; Videira et al. 2016
*Chaetocapnodiumindonesiacum*	Indonesia	*Camelliasinensis*	CBS 202.30	MH855113	GU301849	GU296178	MN829273	GU349060	Schoch et al. 2009; Vu et al. 2019; Abdollahzadeh et al. 2020
*Chaetocapnodiuminsulare*	South Africa	*Phylicaarborea*	CBS 146159	NR_168830	MN749178	–	MN829274	MN829359	Abdollahzadeh et al. 2020
*Chaetocapnodiumphilippinense*	Philippines	palm	MFLUCC 12-0110	NR_168831	KP744503	–	MN829277	MN829362	Liu et al. 2015; Abdollahzadeh et al. 2020
*Chaetocapnodiumplacitae*	Australia	*Eucalyptusplacita*	CBS 124758	GQ303268	GQ303299	–	MN829278	MN829363	Cheewangkoon et al. 2009; Abdollahzadeh et al. 2020
*Chaetocapnodiumsiamensis*	Thailand	leaves of unidentified plant	MFLUCC 13-0778	–	KP744479	–	–	–	Liu et al. 2015
*Chaetocapnodiumsummerellii*	Australia	*Eucalyptusplacita*	CBS 146157	NR_168829	MN749176	–	MN829271	MN829357	Abdollahzadeh et al. 2020
*Chaetocapnodiumtanzanicum*	Tanzania	lichen	CBS 145.79	NR_168832	MN749182	–	MN829280	MN829365	Abdollahzadeh et al. 2020
*Chaetocapnodiumthailandense*	Thailand	–	CBS 139619	NR_168833	MN749183	–	MN829281	MN829366	Abdollahzadeh et al. 2020
*Chaetothyrinaguttulata*	Thailand	*Mangiferaindica*	MFLUCC 15-1080	KX372277	KU358917	KU358916	–	–	Hongsanan et al. 2017
*Chaetothyrinamusarum*	Thailand	*Musa* sp.	MFLUCC 15-0383	KX372275	KU710171	KU710174	–	–	Singtripop et al. 2016; Hongsanan et al. 2017
*Cladosporiumallicinum*	Czech Republic	*Polygonatumodoratum*	CBS 813.71	–	DQ008149	–	–	–	Avila et al. 2005
*Cladosporiumiridis*	Netherlands	*Iris* sp.	CBS 138.40	EU167591	DQ008148	EU167591	KT223022	–	Avila et al. 2005; Simon et al. 2009; Ismail et al. 2016
*Cladosporiumramotenellum*	United Kingdom	leaves of *Arundo* sp.	CBS 170.54	MH857281	DQ678057	DQ678004	DQ677952	DQ677898	Schoch et al. 2006b; Vu et al. 2019
*Comminutisporaagavacearum*	USA	*Dasylirionleiophyllum*	CBS 619.95	MH862543	EU981286	–	MN829337	MN829423	Tsuneda et al. 2008; Vu et al. 2019; Abdollahzadeh et al. 2020
*Conidiocarpusasiaticus*	Thailand	*Coffeaarabica*	MFLUCC 10-0062	KU358924	JN832612	JN832597	–	–	Chomnunti et al. 2011; Hongsanan et al. 2015b
*Conidiocarpuscaucasicus*	Iran	*Citrussinensis*	GUMH 937	–	KC833050	KC833051	–	–	Bose et al. 2014
*Conidiocarpussiamensis*	Thailand	*Mangiferaindica*	MFLUCC 10-0064	–	JN832609	JN832594	–	–	Chomnunti et al. 2011
*Cystocoleusebeneus*	Austria	–	L161	–	EU048578	EU048571	–	–	Muggia et al. 2008
*Cystocoleusebeneus*	Austria	–	L348	–	EU048580	–	–	–	Muggia et al. 2008
*Davidiellomycesaustraliensis*	Australia	leaves of Cyperaceae	CPC 29170	KY979737	KY979792	–	LT799790	–	Crous et al. 2017; Bezerra et al. 2017
*Dissoconiumaciculare*	Germany	*Astragalus* sp.	CBS 204.89	AY725520	GU214419	GU214523	KX288435	–	Crous et al. 2004, 2009b; Videira et al. 2016
*Dissoconiumaciculare*	Netherlands	*Brassica* sp.	CBS 201.89	AY725519	GU214418	GU214522	KT216557	–	Crous et al. 2004, 2009b; Ismail et al. 2016
*Dissoconiumaciculare*	USA	*Malusdomestica*	CBS 132080	JQ622083	JQ622091	–	–	–	Li et al. 2012
*Dissoconiumaciculare*	USA	*Malusdomestica*	CBS 132081	AY598874	JQ622097	–	–	–	Batzer et al. 2005; Li et al. 2012
*Dothideainsculpta*	France	*Clematisvitalba*	CBS 189.58	AF027764	DQ247802	DQ247810	DQ247792	DQ471081	Jacobs & Rehner 1998; Schoch et al. 2006a; Spatafora et al. 2006
*Dothideasambuci*	Austria	*Sambucusnigra*	AFTOL-ID 274	DQ491505	AY544681	AY544722	–	–	Lutzoni et al. 2004; James et al. 2006
*Dothioracannabinae*	India	*Daphnecannabina*	AFTOL-ID 1359	NR_144904	DQ470984	DQ479933	DQ470936	DQ471107	De Hoog et al. 1999; Spatafora et al. 2006
*Dothioraphillyreae*	Spain	*Phillyreaangustifolia*	CBS 473.69	NR_155057	EU754146	EU754047	–	–	de Gruyter et al. 2009; Crous & Groenewald 2016
*Elsinoephaseoli*	Cuba	*Phaseoluslunatus*	AFTOL-ID 1855	NR_148161	DQ678095	DQ678042	KX887144	DQ677935	Schoch et al. 2006b; Fan et al. 2017
*Extremusantarcticus*	Antarctica	rock	CCFEE 5312	KF309979	KF310020	–	–	–	Egidi et al. 2014
*Fumiglobuspieridicola*	Canada	*Pierisjaponica*	UBC F23788	NR_153985	KC833052	NG_065012	–	–	Bose et al. 2014
*Graphiopsischlorocephala*	Germany	*Paeoniadelavayi*	CBS 121522	EU009457	EU009457	–	LT799753	–	Schubert et al. 2007; Bezerra et al. 2017
*Graphiopsischlorocephala*	New Zealand	*Paeonia* sp.	CBS 100405	EU009456	EU009456	–	KT216520	–	Schubert et al. 2007; Ismail et al. 2016
*Heteroconiumcitharexyli*	Ecuador	*Citharexylumilicifolium*	S (type)	HM628776	HM628775	–	–	–	Cheewangkoon et al. 2012
*Hortaeawerneckii*	Greece	sea water-sprayed marble	CBS 100496	AY128703	GU301817	GU296152	GU371739	GU349050	De Leo et al. 2003; Schoch et al. 2009
*Houjiayanglingensis*	China	*Malusdomestica*	CBS 125225	MH863464	GQ433631	–	–	–	Yang et al. 2010; Vu et al. 2019
*Houjiayanglingensis*	China	*Malusdomestica*	CBS 125226	GQ433629	GQ433630	–	–	–	Yang et al. 2010
*Hyalinozasmidiumaerohyalinosporum*	Australia	*Eucalyptustectifica*	CBS 125011	KF901605	KF901930	–	MF951504	–	Quaedvlieg et al. 2014; Videira et al. 2017
*Hyphoconissterilis*	Spain	rock	TRN287	AY843125	KF310032	AY843257	–	_	Ruibal et al. 2008; Egidi et al. 2014
*Leptoxyphiumcacuminum*	Thailand	*Gossypiumherbaceum*	MFLUCC 10-0059	–	JN832603	JN832588	–	–	Chomnunti et al. 2011
*Leptoxyphiumcitri*	Spain	*Citrussinensis*	CBS 451.66	MN749266	KF902094	–	GU371727	GU349039	Schoch et al. 2009; Quaedvlieg et al. 2014; Abdollahzadeh et al. 2020
*Leptoxyphiumglochidion*	China	*Glochidionwrightii*	IFRDCC 2651	NR_155316	KF982308	NG_065036	–	–	Yang et al. 2014
*Leptoxyphiumkurandae*	Australia	*Eucalyptus* sp.	CBS 129530	JF951150	JF951170	–	MN829295	MN829379	Crous et al. 2011a; Abdollahzadeh et al. 2020
*Leptoxyphiummadagascariense*	Madagascar	*Eucalyptuscamaldulensis*	CBS 124766	GQ303277	MH874923	–	MN829296	MN829380	Cheewangkoon et al. 2009; Vu et al. 2019; Abdollahzadeh et al. 2020
*Microcyclosporellamali*	Slovenia	*Malusdomestica*	CBS 126136	MH864045	GU570547	–	KX288436	–	Frank et al. 2010; Videira et al. 2016; Vu et al. 2019
*Mycosphaerelloidesmadeirae*	Netherlands	*Quercusrobur*	CBS 116066	AY853188	KX286989	–	KX288444	–	Videira et al. 2016
*Myriangiumhispanicum*	–	*Acermonspessulanum*	CBS 247.33	MH855426	GU301854	GU296180	GU371744	GU349055	Schoch et al. 2009; Vu et al. 2019
*Neoantennariellaphylicae*	United Kingdom	*Phylicaarborea*	CBS 146163	NR_168834	MN749211	–	MN829313	MN829397	Abdollahzadeh et al. 2020
*Neoasbolisiaphylicae*	United Kingdom	*Phylicaarborea*	CBS 146168	NR_168835	MN749215	–	MN829317	MN829401	Abdollahzadeh et al. 2020
*Neocladosporiumleucadendri*	South Africa	*Leucadendron* sp.	CBS 131317	NR_152324	JQ044455	–	LT799755	–	Crous et al. 2011b; Bezerra et al. 2017
*Neodevriesiahilliana*	New Zealand	*Macrozamiacommunis*	CBS 123187	NR_145098	GU214414	–	LT799761	–	Crous et al. 2009b; Bezerra et al. 2017
*Neodevriesiamodesta*	Italy	rock	CBS 137182	NR_144975	KF310026	–	–	–	Egidi et al. 2014
*Neodevriesiapakbiae*	Thailand	unidentified fern	CBS 139914	NR_137997	KR476775	–	–	–	Crous et al. 2015
*Neodevriesiastirlingiae*	Australia	*Stirlingialatifolia*	CBS 133581	NR_120228	KC005799	–	–	–	Crous et al. 2012
*Neodevriesiastrelitziae*	South Africa	*Strelitzianicolai*	CBS 122379	NR_175123	GU301810	NG_078729	GU371738	GU349049	Arzanlou et al. 2008; Schoch et al. 2009; Vu et al. 2019
*Neodevriesiaxanthorrhoeae*	Australia	*Xanthorrhoeaaustralis*	CBS 128219	NR_144962	HQ599606	–	–	–	Crous et al. 2010
*Neomycosphaerellapseudopentameridis*	South Africa	*Pseudopentamerismacrantha*	CBS 136407	KF777173	KF777226	–	MF951545	–	Crous et al. 2013b; Videira et al. 2017
*Neophaeothecasalicorniae*	South Africa	*Salicornia* sp.	CBS 141299	NR_145401	KX228327	–	MN829343	MN829429	Crous et al. 2016; Abdollahzadeh et al. 2020
*Neophaeothecatriangularis*	Belgium	wet surface of humidifier of air conditioning unit	CBS 471.90	MH862225	EU019279	–	MN829344	MN829430	Crous et al. 2007a; Vu et al. 2019; Abdollahzadeh et al. 2020
*Neoramulariopsiscatenulata*	Rwanda	*Phaseolusvulgaris*	CBS 355.73	NR_153920	KX286973	–	KX288424	–	Videira et al. 2016
*Paradevriesiacompacta*	Spain	rock	CBS 118294	NR_144955	GU323220	NG_064945	GU371751	GU349088	Ruibal et al. 2005, 2009; Schoch et al. 2009
*Paramycosphaerellaintermedia*	New Zealand	*Eucalyptussaligna*	CBS 114356	NR_164413	KF902026	–	–	–	Quaedvlieg et al. 2014; Lee et al. 2016
*Paramycosphaerellamarksii*	South Africa	*Eucalyptusgrandis*	CBS 110750	DQ267596	DQ204757	–	–	–	Hunter et al. 2006
*Penidiella* sp.	–	–	CPC 16707	MN749304	MN749230	–	MN829339	MN829425	Abdollahzadeh et al. 2020
*Petrophilaincerta*	Spain	rock	CBS 118608	NR_144956	KF310030	–	–	–	Ruibal et al. 2005; Egidi et al. 2014
*Phaeothecafissurella*	Canada	*Pinuscontorta*	CBS 520.89	MH862184	GU117900	NG_065804	MN829342	MN829428	Sterflinger et al. 1999; Yang et al. 2010; Vu et al. 2019
*Phaeothecoidiellaillinoisensis*	USA	*Malus* sp.	CBS 125223	NR_137740	GU117901	–	–	–	Yang et al. 2010
*Phaeothecoidiellamissouriensis*	USA	*Malus* sp.	CBS 118959	GU117899	GU117903	–	–	–	Yang et al. 2010
*Phaeoxyphiellaaustraliana*	Australia	*Agonis* sp.	CBS 146169	NR_168837	MN749220	–	MN829322	MN829406	Abdollahzadeh et al. 2020
*Phaeoxyphiellaphylicae*	United Kingdom	*Phylicaarborea*	CBS 146170	NR_168836	MN749219	–	MN829321	MN829405	Abdollahzadeh et al. 2020
*Phloeosporaulmi*	Austria	*Ulmusglabra*	CBS 344.97	KF251202	KF251705	–	–	–	Quaedvlieg et al. 2013
*Phragmocapniasbetle*	Thailand	*Ixora* sp.	MFLUCC 10-0053	KU358922	JN832606	JN832591	–	–	Chomnunti et al. 2011; Hongsanan et al. 2015b
*Phragmocapniasplumeriae*	Thailand	*Plumeria* sp.	MFLUCC 15-0205	KU358919	KU358918	–	–	–	Hongsanan et al. 2015b
*Polychaetoncitri*	Iran	*Citrusaurantium*	CBS 116435	GU214649	GU214469	–	MN829310	MN829394	Crous et al. 2009b; Abdollahzadeh et al. 2020
*Pseudoveronaeaellipsoidea*	USA	*Malusdomestica*	CBS 132085	NR_111367	FJ147154	–	KT921165	–	Diaz Arias et al. 2010; Ismail et al. 2016
*Pseudoveronaeaobclavata*	USA	*Malusdomestica*	CBS 132086	NR_111168	JQ622102	–	–	–	Batzer et al. 2005; Li et al. 2012
*Pseudozasmidiumeucalypti*	Australia	*Eucalyptustereticornis*	CBS 121101	KF901606	KF901931	–	MF951637	–	Quaedvlieg et al. 2014; Videira et al. 2017
*Rachicladosporiumamericanum*	USA	leaf litter	CBS 124774	NR_175021	GQ303323	–	MN829336	MN829421	Cheewangkoon et al. 2009; Abdollahzadeh et al. 2020
*Rachicladosporiumcboliae*	USA	twig	CBS 125424	MH863703	GU214484	NG_062827	LT799763	MN829422	Crous et al. 2009b; Bezerra et al. 2017; Vu et al. 2019; Abdollahzadeh et al. 2020
*Rachicladosporiumeucalypti*	Ethiopia	*Eucalyptusglobulus*	CBS 138900	NR_155718	KP004476	–	–	–	Crous et al. 2014
*Rachicladosporiumpini*	Netherlands	*Pinusmonophylla*	CBS 129525	JF951145	JF951165	–	LT799764	–	Crous et al. 2011a; Bezerra et al. 2017
*Racodiumrupestre*	Austria	–	L346	GU067666	EU048583	EU048575	–	–	Muggia et al. 2008; Muggia & Grube 2010
*Racodiumrupestre*	United Kingdom	–	L423	GU067668	EU048581	–	–	–	Muggia et al. 2008; Muggia & Grube 2010
*Racodiumrupestre*	Italy	–	L424	GU067669	EU048582	–	–	–	Muggia et al. 2008; Muggia & Grube 2010
*Ramichloridiumluteum*	China	*Malusdomestica*	CBS 132088	NR_119684	JQ622099	–	MF951417	–	Li et al. 2012; Videira et al. 2017
*Ramulariaendophylla*	Netherlands	*Quercusrobur*	CBS 113265	KF251220	KF251723	–	–	–	Quaedvlieg et al. 2013
*Ramularianyssicola*	USA	*Nyssaogeche* x *sylvatica*	CBS 127665	NR_111549	NG_070531	–	KJ504636	–	Minnis et al. 2011; Videira et al. 2015a
*Ramulariapusilla*	Germany	*Poaannua*	CBS 124973	NR_154917	KP894141	–	KP894687	–	Videira et al. 2015b
*Readeriellanontingens*	Australia	*Eucalyptusoblonga*	CPC 14444	KF901726	KF902073	–	–	–	Quaedvlieg et al. 2014
*Readerielliopsisfuscoporiae*	French Guiana	*Fuscoporiawahlbergii*	CBS 139900	NR_137978	KR476755	–	MN829326	MN829410	Crous et al. 2015; Abdollahzadeh et al. 2020
*Readerielliopsisguyanensis*	French Guiana	decaying leaf	CBS 117550	NR_176103	FJ493211	–	MN829327	MN829411	Crous et al. 2008; Abdollahzadeh et al. 2020
*Saxophilatyrrhenica*	Italy	stone monument	CCFEE 5935	KP791764	NG_059571	–	–	–	Isola et al. 2016
*Schismatommadecolorans*	–	–	AFTOL-ID 307	AY548808	AY548815	AY548809	DQ883715	DQ883725	Lutzoni et al. 2004; Spatafora et al. 2006
*Schizothyriumcryptogama*	USA	*Malusdomestica*	CBS 125658	FJ425208	FJ147157	–	KT216548	–	Diaz Arias et al. 2010; Ismail et al. 2016
*Schizothyriumpomi*	USA	*Malusdomestica*	CBS 125312	FJ425206	FJ147155	–	KT216539	–	Diaz Arias et al. 2010; Ismail et al. 2016
*Schizothyriumwisconsinensis*	USA	*Malusdomestica*	CBS 125659	FJ425209	FJ147158	–	KT216549	–	Diaz Arias et al. 2010; Ismail et al. 2016
*Scolecoxyphiumblechni*	United Kingdom	*Blechnumpalmiforme*	CBS 146174	NR_168838	MN749224	–	MN829328	MN829412	Abdollahzadeh et al. 2020
*Scolecoxyphiumblechnicola*	United Kingdom	*Blechnumpalmiforme*	CBS 146175	NR_168839	MN749225	–	MN829329	MN829413	Abdollahzadeh et al. 2020
*Scolecoxyphiumleucadendri*	South Africa	*Leucadendron* sp.	CBS 146176	NR_168840	MN749226	–	MN829330	MN829414	Abdollahzadeh et al. 2020
*Scolecoxyphiumphylicae*	South Africa	*Phylicaarborea*	CBS 146177	NR_168841	MN749227	–	MN829331	MN829415	Abdollahzadeh et al. 2020
*Scoriasaphidis*	–	aphid	CBS 325.33	GU214696	MH866910	–	KT216542	MN829417	Crous et al. 2009b; Ismail et al. 2016; Vu et al. 2019; Abdollahzadeh et al. 2020
*Scoriascamelliae*	Indomesia	*Camelliasinensis*	CBS 201.30	MH855112	MH866560	–	MN829333	MN829418	Vu et al. 2019; Abdollahzadeh et al. 2020
*Scoriasleucadendri*	South africa	*Leucadendronmuirii*	CBS 131318	JQ044437	JQ044456	–	MN829334	MN829419	Crous et al. 2011b; Abdollahzadeh et al. 2020
*Scoriasmangiferae*	Thailand	*Mangiferaindica*	MFLUCC 15-0230	NR_154422	KT588603	–	–	–	Hongsanan et al. 2015a
*Scoriasspongiosa*	Thailand	*Entada* sp.	MFLUCC 10-0084	–	JN832601	JN832586	–	–	Chomnunti et al. 2011
*Septorialycopersici*	South Korea	*Lycopersiconesculentum*	CBS 128654	MH865102	KF251966	–	KX348091	–	Verkley et al. 2013; Videira et al. 2016; Vu et al. 2019
*Septoriaprotearum*	South Africa	*Zantedeschiaaethiopica*	CBS 135477	KF251524	KF252029	–	–	–	Verkley et al. 2013
*Sporidesmajorapennsylvaniensis*	USA	*Malusdomestica*	CBS 125229	NR_156639	MF951122	–	MF951424	–	Videira et al. 2017
*Stomiopeltisversicolor*	USA	*Malusdomestica*	GA3-23C2b	FJ438375	FJ147163	–	–	–	Diaz Arias et al. 2010
*Teratosphaeriastellenboschiana*	South Africa	*Eucalyptuspunctata*	CBS 125215	KF901733	KF937247	–	–	–	Quaedvlieg et al. 2014
*Teratosphaeriaceae* sp.	–	–	CPC 16695	MN749303	MN749231	–	MN829340	MN829426	Abdollahzadeh et al. 2020
*Teratosphaeriaceae* sp.	–	–	CPC 17588	MN749305	MN749232	–	MN829341	MN829427	Abdollahzadeh et al. 2020
*Uwebrauniacommune*	South Africa	*Eucalyptusnitens*	CBS 110747	AY725535	GU214420	GU214525	KT216558	–	Crous et al. 2004, 2009b; Ismail et al. 2016
*Verrucocladosporiumdirinae*	United Kingdom	*Dirinamassiliensis*	CBS 112794	EU040244	EU040244	–	–	–	Crous et al. 2007b
*Xenodevriesiastrelitziicola*	South Africa	*Strelitzia* sp.	CBS 122480	NR_171741	NG_059085	–	–	–	Crous et al. 2009b; Vu et al. 2019
*Xenomycosphaerellaelongata*	Venezuela	*Eucalyptuscamaldulensis* x *urophylla*	CBS 120735	NR_154469	JF700942	–	MF951687	–	Crous et al. 2007c; Quaedvlieg et al. 2011; Videira et al. 2017
*Zasmidiumpseudotsugae*	USA	*Pseudotsugamenziesii*	rapssd	EF114687	EF114704	EF114729	–	–	Winton et al. 2007
*Zasmidiumtsugae*	USA	*Tsugaheterophylla*	ratstk	EF114688	EF114705	EF114730	–	–	Winton et al. 2007

AFTOL-ID: Assembling the Fungal Tree of Life (AFTOL); CBS: Westerdijk Fungal Biodiversity Institute, Utrecht, the Netherlands; CCFEE = Culture Collection of Fungi from Extreme Environments, Tuscia University, Viterbo, Italy; CPC: Culture collection of Pedro Crous, housed at Westerdijk Fungal Biodiversity Institute, Utrecht, the Netherlands; GUMH: Guilan University Mycological Herbarium, Rasht, Iran; IFRDCC: International Fungal Research & Development Centre Culture Collection, Chinese Academy of Forestry, Kunming, China; MFLUCC: Mae Fah Luang University Culture Collection, Chiang Rai, Thailand; S: Herbarium of the Swedish Museum of Natural History, Stockholm, Sweden; UBC: Herbarium of the University of British Columbia, Vancouver, Canada. Unknown abbreviations: E, GA3-23C2b, L, MA, rapssd, ratstk, TRN – indicates unavailable data or sequence.

For phylogenetic analyses, sequences were separately aligned for each single-gene dataset using MAFFT algorithm (Katoh et al. 2005) in Geneious 11.1.5. The phylogenetic reconstructions were performed using the concatenated ITS-LSU-SSU-*rpb2*-*tef1* alignment. Maximum likelihood (ML) analysis was conducted using RAxML-NG v. 1.1.1 (Kozlov et al. 2019), with a bootstrap of 1000 replicates. Bayesian inference (BI) analysis was carried out using MrBayes v. 3.2.6 (Ronquist et al. 2012). For both ML and BI analyses, the ModelTest-NG v. 0.2.0 was used to select the best substitution models using Bayesian Information Criterion (BIC) (Darriba et al. 2020). BI analysis was performed by running 2 000 000 generations in four chains, saving the current tree to file every 100 generations. The first 25% of trees were discarded as burn-in. Average standard deviations of split frequencies were <0.01 at the end of the runs. The final phylogenetic trees were visualized using FigTree v1.4.3. The alignment was deposited at figshare.com (https://doi.org/10.6084/m9.figshare.25623660.v1).

## ﻿Results

### ﻿Phylogenetic analyses

**Figure 1.**
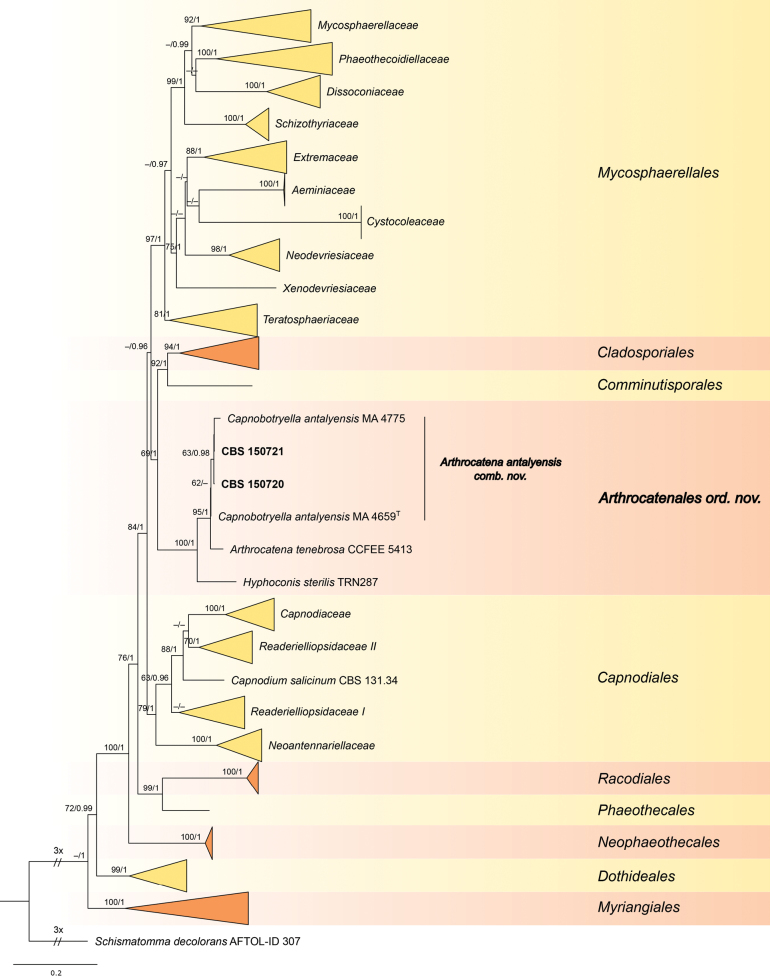
Reduced phylogenetic tree of selected members of the Capnodiales s. lat., Dothideales and Myriangiales, including all described species of the genera *Arthrocatena* and *Hyphoconis*, obtained from a maximum likelihood analysis of the combined multi-locus alignment (ITS, LSU, SSU, *rpb2*, *tef1*). The positions of new strains, *Arthrocatenaantalyensis* comb. nov. and new order Arthrocatenales are indicated in **bold**. Ex-type cultures are indicated with superscript T. Numbers above branches indicate maximum likelihood bootstrap (MLB) support values > 60% and Bayesian posterior probabilities (BPP) > 0.95, respectively (MLB/BPP). *Schismatommadecolorans* was used as an outgroup. The scale bar represents the expected number of changes per site.

The concatenated ITS-LSU-SSU-*rpb2*-*tef1* alignment contained sequences belonging to 130 species, including *Schismatommadecolorans* used as an outgroup. The alignment comprised a total of 4829 characters (ITS: 829, LSU: 819, SSU: 946, *rpb2*: 1122, *tef1*: 1113), including alignment gaps. The best matching substitution models selected for single locus alignments in the ML analysis were as follows: GTR+I+G4 for ITS, TIM3+I+G4 for LSU, K80+I+G4 for SSU, TIM2+I+G4 and TPM3uf+I+G4 for *rpb2* (three codons), and JC+I+G4, HKY+I+G4 and TIM3+I+G4 for *tef1* (three codons). The BI analysis was performed with the following substitution models: GTR+I+G4 for ITS and LSU, K80+I+G4 for SSU, GTR+I+G4 and HKY+I+G4 for *rpb2* codons, and JC+I+G4, HKY+I+G4 and HKY+G4 for *tef1* codons. ML and BI analyses resulted in similar tree topologies (Suppl. materials 1, 2). The best scoring maximum likelihood phylogenetic tree is shown on Fig. 1. Maximum likelihood bootstrap (MLB) support values above 60% and Bayesian posterior probabilities (BPP) above 0.95 are shown at the nodes.

The sequences of analyzed strains CBS 150720 and CBS 150721 clustered with sequences of type and additional strain of *Capnobotryellaantalyensis* in a moderately supported clade (only in ML: MLB = 62%), which was sister to *Arthrocatenatenebrosa* with high support (MLB = 95%, BPP = 1). *Capnobotryellaantalyensis* is recombined here to *Arthrocatena*. The sequence similarity between different strains of *Arthrocatenaantalyensis* ranges between 97.4% and 98.7% in ITS. The sequence similarity between *A.antalyensis* and *A.tenebrosa* is 96.1–96.9% in ITS. Members of *Arthrocatena* formed a fully supported sister clade to single species lineage representing the genus and species *Hyphoconissterilis*. The clustering of *Arthrocatena* + *Hyphoconis* was resolved at sister position (MLB = 69%, BPP = 1) to clades representing orders Cladosporiales and Comminutisporales. The new order Arthrocatenales and new family Arthrocatenaceae are proposed to this clade.

### ﻿Taxonomy

#### ﻿ Arthrocatenales

Taxon classificationFungiDothideomycetesArthrocatenales

Piątek, Stryjak-Bogacka & Czachura ord. nov.

7DC36CED-6119-5ACA-9F07-299205F5FDE5

854789

##### Etymology.

Named after the genus *Arthrocatena*.

##### Description.

Colonies erumpent, spreading, with elevated and folded center, greenish olivaceous, forming concentric rings, margin smooth, entire or undulate. Reverse black. Mycelium composed of branched, septate, pale brown or brown, smooth, straight, flexuose or torulose, thin-walled hyphae. Arthroconidia ellipsoid or broadly ellipsoid, rarely barrel-shaped, brown, smooth, one-septate, intercalary or on side branches, single or in chains. Chlamydospore-like cells spherical, brown, smooth, aseptate, intercalary, in simple or branched chains. Chlamydospores spherical, brown, smooth, muriformly septate, intercalary, single.

##### Type family.

Arthrocatenaceae Piątek, Stryjak-Bogacka & Czachura.

#### ﻿ Arthrocatenaceae

Taxon classificationFungiArthrocatenalesArthrocatenaceae

Piątek, Stryjak-Bogacka & Czachura fam. nov.

3046409E-AFC1-5F9A-A2AF-385807D2E6F3

854790

##### Etymology.

Named after the genus *Arthrocatena*.

##### Description.

Colonies erumpent, spreading, with elevated and folded center, greenish olivaceous, forming concentric rings, margin smooth, entire or undulate. Reverse black. Mycelium composed of branched, septate, pale brown or brown, smooth, straight, flexuose or torulose, thin-walled hyphae. Arthroconidia ellipsoid or broadly ellipsoid, rarely barrel-shaped, brown, smooth, one-septate, intercalary or on side branches, single or in chains. Chlamydospore-like cells spherical, brown, smooth, aseptate, intercalary, in simple or branched chains. Chlamydospores spherical, brown, smooth, muriformly septate, intercalary, single.

##### Type genus.

*Arthrocatena* Egidi & Selbmann.

#### ﻿ Arthrocatena antalyensis

Taxon classificationFungiArthrocatenalesArthrocatenaceae

(Sert & Sterfl.) Piątek, Stryjak-Bogacka & Czachura comb. nov.

014183E8-D106-5ADF-B217-6DC3228A1762

854791

Figs 2 , 3 , 4

##### Basionym.

*Capnobotryellaantalyensis* Sert & Sterfl., Mycol. Res. 111(10): 1237 (2007).

##### Typus.

Turkey, Antalya, isolated from the surface of a child’s grave in Side museum (holotype: ACBR MA 4659).

##### DNA barcodes (from analysed strains).

ITS (OR096278, OR096279), LSU (OR096282, OR096283), SSU (OR096280, OR096281), *rpb2* (OR096699, OR096700).

##### Description.

Mycelium composed of branched, septate, pale brown or brown, smooth, straight, flexuose or torulose, thin-walled hyphae, 3.5–7.0 µm wide, consisting of elongated, subglobose, broadly ellipsoidal or pyriform cells, sometimes anastomosing; hyphae develop into arthroconidia, chlamydospore-like cells or chlamydospores. Arthroconidia ellipsoid or broadly ellipsoid, rarely barrel-shaped, brown, smooth, one-septate, 9.0–19.0(–23.0) × 6.5–8.5 µm, produced intercalary or rarely on side branches, single or in chains. Chlamydospore-like cells spherical, brown, smooth, aseptate, 7.0–12.0 × 7.0–10.0 µm, produced intercalary, in simple or branched chains. Chlamydospores spherical, brown, smooth, muriformly septate, 12.5–15.0 × 11.0–14.0 µm, produced intercalary, single between chlamydospore-like cells.

**Figure 2.**
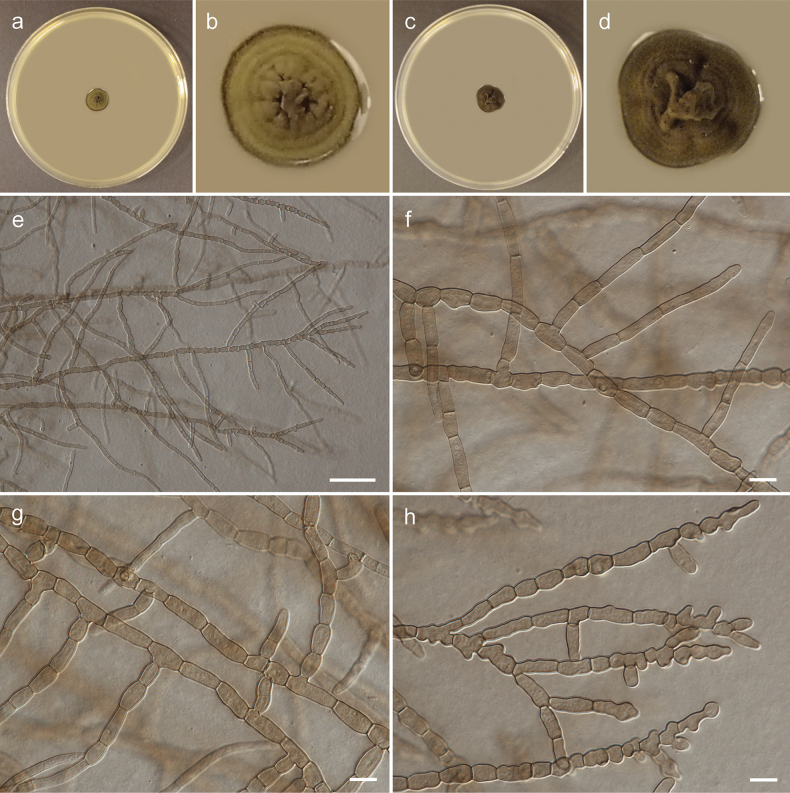
Morphology of *Arthrocatenaantalyensis* (strain CBS 150720, **e–h** slide culture on PDA): **a, b** general view and detailed view of upper side of colony on MEA after 4 weeks of growth at 25 °C **c, d** general view and detailed view of upper side of colony on PDA after 4 weeks of growth at 25 °C **e** general view of hyphae **f, g** straight hyphae (note anastomosing hyphae visible on figure g) **h** terminal, flexuose and torulose hyphae. Scale bars: 50 µm (**e**); 10 µm (**f–h**).

##### Culture characteristics.

Colonies on MEA erumpent, spreading, with elevated and folded center, greenish olivaceous, forming concentric rings, reaching 8 mm diam after 4 weeks growth at 15 °C and 12 mm diam after 4 weeks growth at 25 °C, surface with moderate aerial mycelium, margin smooth and entire, darker than the remaining part. Reverse black. Colonies on PDA erumpent, spreading, with elevated and folded center, greenish olivaceous, forming indistinct concentric rings, reaching 10 mm diam after 4 weeks growth at 15 °C and 14 mm diam after 4 weeks growth at 25 °C, surface with sparse aerial mycelium, margin smooth and undulate, concolours with the remaining part. Reverse black.

**Figure 3.**
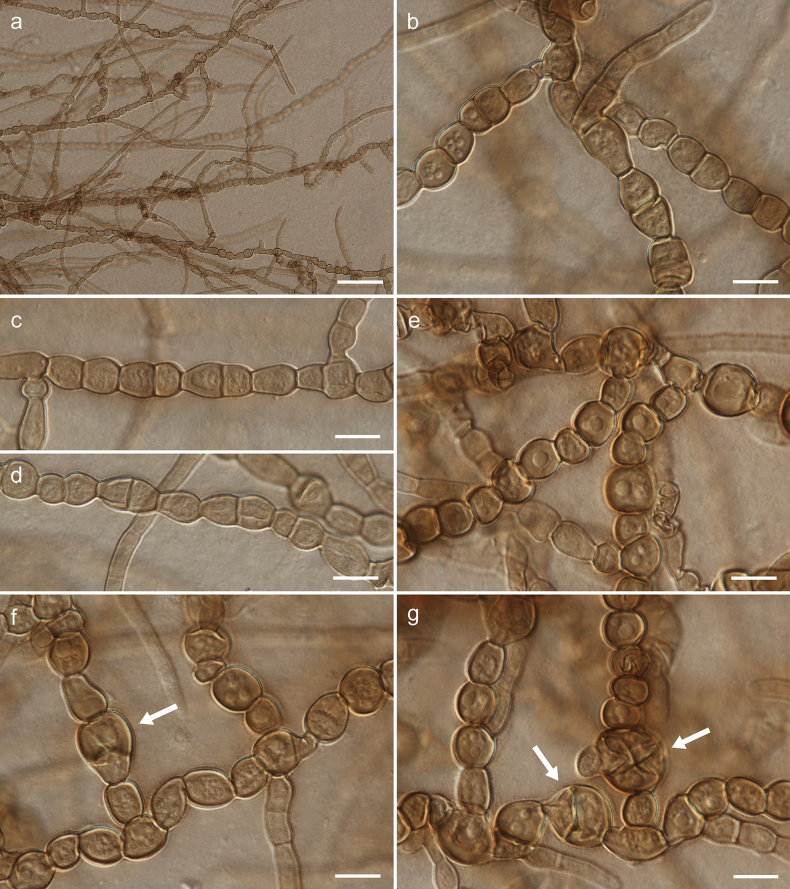
Morphology of *Arthrocatenaantalyensis* (strain CBS 150720, slide culture on PDA): **a** general view of hyphae, arthroconidia and chlamydospore **b–d** arthroconidia **e** chlamydospore-like cells **f–g** chlamydospore-like cells and muriformly septate chlamydospores (indicated by arrows). Scale bars: 50 µm (**a**); 10 µm (**b–g**).

##### Specimens examined.

Poland, Silesian Province, Katowice County: Katowice-Bogucice, municipal greenery, isolated from sooty mould community on *Tiliacordata* leaves, 10 Sept. 2018, leg. M. Piątek, W. Bartoszek & P. Czachura (KRAM F-59837; culture: G57 = CBS 150720); Podkarpackie Province, Rzeszów County: Rzeszów–Generała Władysława Andersa, municipal greenery, isolated from sooty mould community on *Pinusnigra* needles, 17 Sept. 2018, leg. M. Piątek, W. Bartoszek & P. Czachura (KRAM F-59838; culture: G385 = CBS 150721).

**Figure 4.**
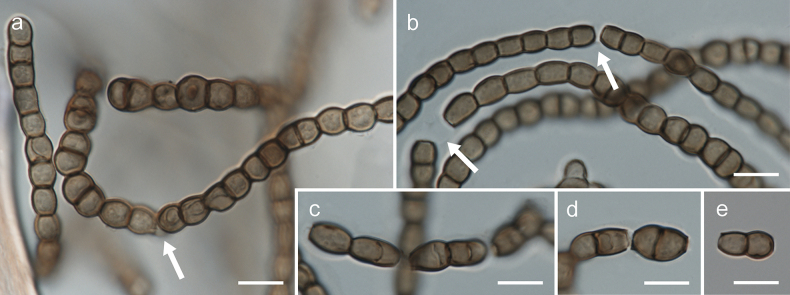
Morphology of *Arthrocatenaantalyensis* (strain CBS 150720, on MEA): **a, b** arthroconidia, arrows indicate disintegrating chains of arthroconidia **c–e** disintegrated arthroconidia. Scale bars: 10 µm (**a–e**).

##### Notes.

*Arthrocatenaantalyensis* differs from *Arthrocatenatenebrosa* in having larger arthroconidia (6.0–11.5 × 3.0–5.5 μm in *A.tenebrosa*; Egidi et al. 2014) and formation of chlamydospore-like cells and muriformly septate chlamydospores.

## ﻿Discussion

Sooty moulds and communities formed by these fungi are still understudied and for that reason they are probably a rich source of interesting or undescribed species. Here, two *Arthrocatena* strains isolated from sooty mould communities on leaves of *Tiliacordata* and needles of *Pinusnigra* in southern Poland were analyzed. Interestingly, in the multi-locus phylogenetic analyses the sequences of the sooty mould strains grouped together with sequences of type and additional strain of *Capnobotryellaantalyensis* (MA 4659 and MA 4775), a rock-inhabiting fungus described from two sites in Turkey (Sert et al. 2007). Support for branch uniting four strains of *C.antalyensis* is moderate and genetic differences between them are relatively high but they are assigned to the same species due to similarities in their micromorphological features. Apart from morphological differences *Capnobotryellaantalyensis* is well separated phylogenetically from its sister species *Arthrocatenatenebrosa*. The affinity of *C.antalyensis* to *A.tenebrosa* was previously shown by Laichmanová (2023) on the phylogenetic tree resolving position of some rock-inhabiting fungal strains from Antarctica. On the other hand, on the phylogenetic trees published by Zucconi et al. (2012), Isola et al. (2016) and De Leo et al. (2022) three strains assigned to *Capnobotryellaantalyensis* (MA 4615, MA 4624, MA 4766) formed a distinct lineage within the current genus *Neodevriesia*. However, none of these strains were originally cited in the protologue of *C.antalyensis* (Sert et al. 2007) and represent other rock-inhabiting fungus, probably undescribed species of *Neodevriesia*. The type species of the genus *Capnobotryella*, *C.renispora*, is a member of the family Teratosphaeriaceae in the Mycosphaerellales (Crous et al. 2009b; Delgado et al. 2018; Li et al. 2020; this study). Therefore, *Capnobotryellaantalyensis* is reallocated to the genus *Arthrocatena*.

The phylogenetic placement of *Arthrocatena* and its sister genus *Hyphoconis* remained unclear. In a study of Egidi et al. (2014), where these two genera were described, they formed a distinct clade within the order Capnodiales s. lat. that was positioned either as sister to clade now assigned to the order Mycosphaerellales or as sister to clades representing current orders Cladosporiales and Mycosphaerellales. In a study of Hongsanan et al. (2020) the genus *Hyphoconis* was placed as sister to clade now assigned to the order Cladosporiales. Consequently, *Arthrocatena* and *Hyphoconis* were included in Capnodiales incertae sedis when using wide concept of the order (Wijayawardene et al. 2014, 2017; Hongsanan et al. 2020) or Mycosphaerellales incertae sedis when using current concept of the capnodialean orders (Wijayawardene et al. 2022).

Our molecular phylogenetic analyses of the concatenated ITS-LSU-SSU-*rpb2*-*tef1* alignment showed that *Arthrocatena* and *Hyphoconis* form a distinct lineage sister to orders Cladosporiales and Comminutisporales. Therefore, a new order Arthrocatenales is described to accommodate these two genera. These three orders have some ecological and morphological peculiarities that differentiate them. Cladosporiales accommodates hundreds of species that are mostly saprobic, rarely lichenicolous, endolithic, endophytic or plant parasitic and distributed over the whole world. They usually produce solitary conidiophores with chains of pigmented conidia, which germinate and grow very quickly on culture media (Abdollahzadeh et al. 2020). Sexual morph is rarely observed in Cladosporiales but, if present, is mycosphaerella-like with pseudothecial ascomata and one-septate ascospores (Bensch et al. 2012; Crous et al. 2014, 2017). Comminutisporales includes only one species, *Comminutisporaagavacearum* (with its asexual morph known as *Hyphosporaagavacearum*), which inhabits dead leaves of *Dasylirionleiophyllum* and *Nolina* sp. (Asparagaceae) in Texas and New Mexico, USA. In the sexual stage it produces pseudothecial, uniloculate ascomata and muriformly septate ascospores, while in the asexual stage it forms hyphae with cellular clumps containing numerous endoconidia (Ramaley 1996; Zalar et al. 1999; Abdollahzadeh et al. 2020). Newly described order Arthrocatenales includes only two genera and three species that are extremophilic fungi isolated from rocks or sooty mould communities. All described species in the Arthrocatenales are known only from sterile mycelia (*Hyphoconis*) that also produce arthroconidia and chlamydospores (*Arthrocatena*).

*Arthrocatena* has been reported, in different and mostly metabarcoding studies, from gut of feather mites in Spain (Doña et al. 2019), plants in China, Estonia and Italy (Wu et al. 2019; Giampetruzzi et al. 2020; Küngas et al 2020), indoor dust in the USA (Cox et al. 2022), tsetse fly in Tanzania (Kim et al. 2022) or rocks in Antarctica (Laichmanová 2023). This suggests that the ecological spectrum and distribution of *Arthrocatenales* may be wider than currently known. However, cultures and multi-locus phylogenetic analyses are necessary to resolve species assignments of fungi detected in the metabarcoding studies.

## Supplementary Material

XML Treatment for Arthrocatenales

XML Treatment for Arthrocatenaceae

XML Treatment for Arthrocatena antalyensis

## ﻿Additional information

### Author ORCIDs

Marcin Piątek https://orcid.org/0000-0002-4968-2861

Monika Stryjak-Bogacka https://orcid.org/0000-0003-2845-9975

Paweł Czachura https://orcid.org/0000-0002-3562-8776

### Data availability

The data that support the findings of this study are available in GenBank (https://www.ncbi.nlm.nih.gov/genbank/) and in culture collections and fungal herbarium, as shown in Table 1 and the text.

## Supplementary materials

Supplementary material 1 Phylogenetic tree of selected members of the Capnodiales s. lat., Dothideales and Myriangiales obtained from a maximum likelihood analysis of the combined multi-locus alignment (ITS, LSU, SSU, *rpb2*, *tef1*) Data type tif Explanation note The positions of new strains, *Arthrocatenaantalyensis* comb. nov. and new order Arthrocatenales are indicated in bold. *Schismatommadecolorans* was used as an outgroup. The scale bar represents the expected number of changes per site. https://binary.pensoft.net/file/1118897This dataset is made available under the Open Database License (http://opendatacommons.org/licenses/odbl/1.0/). The Open Database License (ODbL) is a license agreement intended to allow users to freely share, modify, and use this Dataset while maintaining this same freedom for others, provided that the original source and author(s) are credited.Marcin Piątek, Monika Stryjak-Bogacka, Paweł Czachura

Supplementary material 2 Phylogenetic tree of selected members of the Capnodiales s. lat., Dothideales and Myriangiales obtained from a Bayesian inference analysis of the combined multi-locus alignment (ITS, LSU, SSU, *rpb2*, *tef1*) Data type tif Explanation note The positions of new strains, *Arthrocatenaantalyensis* comb. nov. and new order Arthrocatenales are indicated in bold. *Schismatommadecolorans* was used as an outgroup. The scale bar represents the expected number of changes per site. https://binary.pensoft.net/file/1118898This dataset is made available under the Open Database License (http://opendatacommons.org/licenses/odbl/1.0/). The Open Database License (ODbL) is a license agreement intended to allow users to freely share, modify, and use this Dataset while maintaining this same freedom for others, provided that the original source and author(s) are credited.Marcin Piątek, Monika Stryjak-Bogacka, Paweł Czachura
